# Sensitivity to haptic sound-localisation cues

**DOI:** 10.1038/s41598-020-79150-z

**Published:** 2021-01-11

**Authors:** Mark D. Fletcher, Jana Zgheib, Samuel W. Perry

**Affiliations:** 1 University of Southampton Auditory Implant Service, University of Southampton, University Road, Southampton, SO17 1BJ UK; 2 Faculty of Engineering and Physical Sciences, University of Southampton, University Road, Southampton, SO17 1BJ UK

**Keywords:** Auditory system, Translational research, Neuroscience, Medical research, Psychology, Human behaviour

## Abstract

Hearing aid and cochlear implant (CI) users often struggle to locate and segregate sounds. The dominant sound-localisation cues are time and intensity differences across the ears. A recent study showed that CI users locate sounds substantially better when these cues are provided through haptic stimulation on each wrist. However, the sensitivity of the wrists to these cues and the robustness of this sensitivity to aging is unknown. The current study showed that time difference sensitivity is much poorer across the wrists than across the ears and declines with age. In contrast, high sensitivity to across-wrist intensity differences was found that was robust to aging. This high sensitivity was observed across a range of stimulation intensities for both amplitude modulated and unmodulated sinusoids and matched across-ear intensity difference sensitivity for normal-hearing individuals. Furthermore, the usable dynamic range for haptic stimulation on the wrists was found to be around four times larger than for CIs. These findings suggest that high-precision haptic sound-localisation can be achieved, which could aid many hearing-impaired listeners. Furthermore, the finding that high-fidelity across-wrist intensity information can be transferred could be exploited in human–machine interfaces to enhance virtual reality and improve remote control of military, medical, or research robots.

## General introduction

Users of hearing-assistive devices, such as hearing aids, bone-conduction hearing systems, and cochlear implants (CIs), typically have a limited ability to locate and segregate sounds^1–4^. This can result in an inability to detect and decipher sound sources in complex acoustic scenes, such as classrooms, social spaces, and busy workplaces. Recently, researchers have provided sound information through haptic stimulation on the wrists or forearm to supplement the electrical CI signal^5^. This “electro-haptic” stimulation^4^ has been shown to substantially improve sound localisation^1,6^ and segregation^2,4,7^, as well as discrimination of more basic sound properties such as pitch^8^. In studies showing benefits to sound localisation and segregation, haptic signals have been extracted from the audio received by hearing-assistive devices behind each ear and delivered to each corresponding wrist^1,2,6^. After only minimal training using this approach, CI users had dramatically improved sound-localisation accuracy, which was similar to that of bilateral hearing-aid users^1,6^. Using the same approach, haptic stimulation was found to improve speech-reception thresholds in spatially-separated noise by around 3 dB^2^.

The dominant spatial hearing cues are time and intensity differences across the ears (interaural time and intensity differences). In previous electro-haptic stimulation studies, both interaural time (ITD) and intensity (IID) difference cues were delivered to the wrists^1,2,6^. However, it was unclear whether one or both cues were used to locate and separate sounds. It was also unclear whether the effects measured in these studies represent the performance limits, or whether still more impressive performance might be achieved after long-term training.

In the current study, two experiments were conducted to establish the sensitivity of the wrists to spatial-hearing cues. The first experiment had three aims. The first was to determine the smallest detectable vibrotactile intensity (TID) and time (TTD) difference across the wrists to establish which cues can be used for haptic sound-localisation. This will be important for determining the prioritisation of cues in haptic signal-processing strategies. The second aim was to determine whether sensitivity differs across stimulation frequencies that might be used in haptic devices, which will be important for informing haptic motor selection. Finally, the third aim was to establish whether TID and TTD sensitivity changes with age. If a decline with age was found, this would reduce the utility of haptic stimulation for aiding older hearing-impaired listeners.

The second experiment also had three aims. The first was to establish whether the results of experiment 1 could be generalised across a range of stimulation intensities that might be used by a haptic device. The second aim was to compare sensitivity for signals both with and without amplitude modulation applied. This is important, as studies showing that haptic stimulation on the wrists can improve speech-in-noise and sound-localisation performance in CI users have modulated the amplitude of the haptic signal using the speech amplitude envelope^1,2,4,6,7^. The tactile system is known to be highly sensitive to amplitude envelope differences^9^. This sensitivity might also be exploited in human–machine interfaces. Finally, the third aim was to determine the dynamic range available for haptic stimulation at the wrists. This would establish whether the large IIDs for more lateral source positions^6^ can be represented and whether additional absolute intensity information might be provided to CI users, whose dynamic range is severely limited^10,11^.

In addition to having strong implications for enhancement of spatial hearing in hearing-impaired listeners, high sensitivity to across-wrist TIDs and TTDs could be exploited in a wide range of human–machine interfaces. Haptic stimulation on the wrists or hands has, for example, been used to enhance the experience of virtual and augmented reality^12,13^, for remote control of laboratory measurement systems^14^, and in guidance systems for human–robot teams^15^. In a medical context, applications of wrist-based haptic systems include rehabilitation after wrist injury^16^, needle guidance^17^, and surgical training^18^. If TID or TTD sensitivity were found to be high, outcomes for any of these applications might be improved by transmitting fine-grain information through differences across the wrists. Furthermore, as for enhancement of spatial hearing, the dependence of sensitivity on age is important for any of the potential applications listed, which all include operators across a wide age range.

## Experiment 1: cue type, stimulation frequency, and age

### Introduction

Experiment 1 will establish across-wrist TID and TTD sensitivity for both young and older participants. Most previous studies of tactile sensitivity have focused on the fingertips. However, the fingertips were not considered a suitable site for a haptic device as they are used in numerous everyday tasks. Instead, this study focused on the wrist as wrist-worn devices are commonplace and so are aesthetically acceptable, do not impede everyday tasks, and have been used in previous studies for enhancing listening using haptics^1,2,4,6^.

While previous studies have not examined TID sensitivity across the wrists, they have shown that the tactile system is highly sensitive to changes in intensity over time at a single site. For example, just-noticeable intensity differences of around 1.5 dB have been found for a 250-Hz sinusoid on the hand (thenar eminence)^19^ and for a 160-Hz sinusoid on the index finger^20^. There is evidence that similar, or perhaps even better, single-site intensity discrimination can be achieved on the wrist^21^. The tactile sensitivity to intensity changes is comparable to intensity discrimination for sequential sounds in a single ear, where just-noticeable differences are between around 0.75 and 1.5 dB^22–24^. In the healthy auditory system, IID sensitivity is similar^25^. For hearing aid users, the just-noticeable IID is typically increased to ~ 2–3 dB^26^ and, for CI users, to ~ 4 dB^27^. However, it should be noted that variance between individuals is high (particularly for CI users) and that most CI users are implanted in only one ear and so have little or no access to IIDs^28^. If the tactile system is shown to be sensitive to TIDs of less than around 2 dB, this would suggest that TID cues could be exploited to enhance spatial hearing for a wide range of hearing-impaired listeners.

Previous studies have not investigated TTD discrimination across the wrists, but some have measured simultaneity detection across the hands. In these studies, which aimed to assess interhemispheric transfer time, a stimulus was presented to a single finger on each hand. Participants were asked to judge whether the stimuli were simultaneous or successive. Simultaneity thresholds of around 30–50 ms were found in young adults (20–40 years)^29,30^. This is several orders of magnitude less sensitive than ITD sensitivity in normal-hearing listeners. However, in this previous work participants were asked to determine whether stimuli were simultaneous for a single presentation. In contrast, the current study measured participants’ ability to discriminate two consecutive presentations, one simultaneous and one non-simultaneous. Our forced-choice discrimination paradigm is expected to be more sensitive for determining detection thresholds^31–33^. For ITDs, normal-hearing listeners have just-noticeable difference thresholds of between 10 and 20 μs^34,35^. For hearing-aid users, thresholds are ~ 50 μs on average^26^, and for CI users that have access to ITD cues, thresholds are ~ 400 μs^27^ (although the variation between individuals is large). Note that the maximum difference in arrival time between the ears is around 650 μs, and so even CI users with access to ITD cues often cannot fully exploit them for sound localisation. Sensitivity to TTDs of less than around 50 μs would be required to have utility for enhancing spatial hearing in a significant number of hearing-impaired listeners.

Sensitivity to various stimulus features is known to be affected by age in both the tactile and auditory systems. As in hearing^36^, detection thresholds increase with age in the tactile system, particularly for higher frequency stimuli^37–39^. Detection thresholds for a 250-Hz sinusoid increase by around 10 dB between the ages of 20 and 50 years, and by around 15 dB between 30 and 60 years^37^. Substantial increases in across-hand simultaneity thresholds have also been found with age, with thresholds more than doubling for older (aged 61 to 80 years) compared to younger (aged 20–40 years) participants^30^. However, aging has not been found to affect single-site intensity difference discrimination^40^. It may therefore be expected that sensitivity to TIDs will be robust to aging, but that TTD sensitivity will be reduced in older participants.

In this experiment, thresholds for discrimination of TIDs and TTDs across the palmer surfaces of each wrist were measured both in young adults (aged 20–30 years) and older adults (aged 50–60 years). Thresholds were measured for sinusoidal stimuli with frequencies of 100 or 250 Hz. These frequencies were selected as the lowest operating frequency of micromotors is typically around 100 Hz and the majority of micromotors have operating frequencies of around 250 Hz (where the tactile system is most sensitive^41^).

### Results

**Figure 1:**
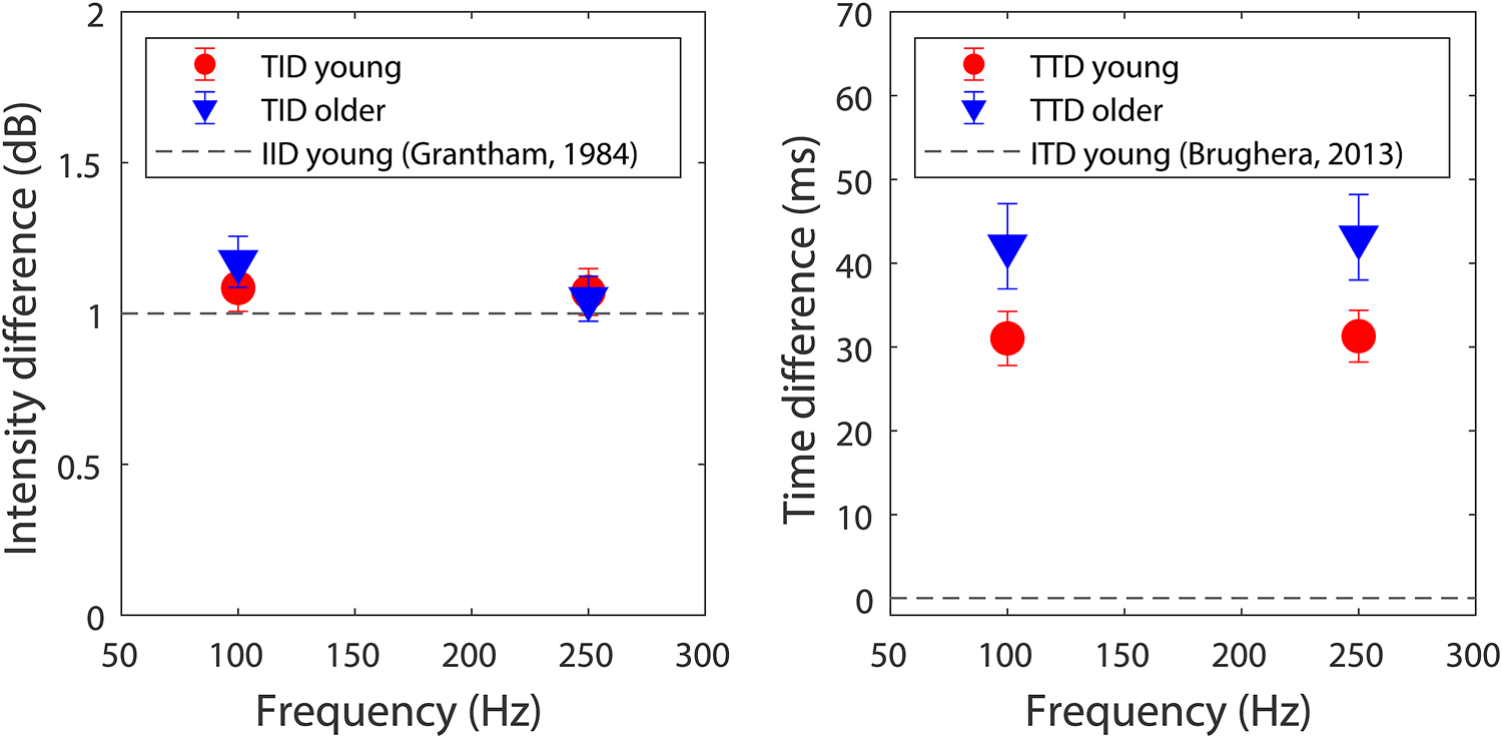
Tactile intensity (TID; left panel) and time (TTD; right panel) difference discrimination thresholds across the wrists for young (red circles) and older (blue downward facing triangles) participants. Thresholds are shown for both 100- and 250-Hz sinusoidal stimuli. For comparison, dashed grey lines show thresholds for interaural intensity (IID) and time (ITD) difference discrimination in young adults. Error bars show the standard error of the mean. This figure was generated using MATLAB R2019a (http://www.mathworks.com/products/matlab/).

Figure 1 shows TID and TTD discrimination thresholds (left and right panels respectively) for the 22 young and 14 older participants who took part in this study. For TIDs, no effect of stimulation frequency (*F*(1,34) = 1.521, *p* = 0.226) or age group (*F*(1,34) = 0.097, *p* = 0.758) was found. The interaction between these factors was also not significant (*F*(1,34) = 0.983, *p* = 0.328). For young participants, the average TID discrimination threshold was 1.08 dB for a 100-Hz stimulus (ranging from 0.38 to 1.75 dB; standard deviation (SD) = 0.36 dB) and 1.07 dB for a 250-Hz stimulus (ranging from 0.54 to 2.04 dB; SD = 0.36 dB). For older participants, the average TID discrimination threshold was 1.17 dB for a 100-Hz stimulus (ranging from 0.52 to 1.65 dB; SD = 0.32 dB) and 1.05 dB for a 250-Hz stimulus (ranging from 0.67 to 1.67 dB; SD = 0.28 dB). Half of the young participants had thresholds (averaged across the two frequencies) below 1 dB and four of the 14 older participants had thresholds below 1 dB.

For TTDs, there was no effect of stimulation frequency (*F*(1,34) = 0.337, *p* = 0.565). However, there was a significant effect of age group (*F*(1,34) = 4.224, *p* = 0.048). The interaction between these factors was not significant (*F*(1,34) = 1.24, *p* = 0.727). For young participants, the average TTD discrimination threshold was 31.03 ms for a 100-Hz stimulus (ranging from 12.76 to 87.5 ms; SD = 15.12 ms) and 31.29 ms for a 250-Hz stimulus (ranging from 13.96 to 82.92 ms; SD = 14.49 ms). For older participants, average TTD discrimination thresholds were 42.03 ms for a 100-Hz stimulus (ranging from 13.96 to 80.00 ms; SD = 19.04 ms) and 43.10 ms for a 250-Hz stimulus (ranging from 10.21 to 83.33 ms; SD = 19.09 ms).

Post-hoc analyses were performed to explore whether TID discrimination thresholds were predictive of TTD discrimination thresholds and whether these thresholds could be predicted by tactile sensitivity or sex. TID and TTD discrimination thresholds were averaged across the two stimulation frequencies and no correction for multiple comparisons was applied for these exploratory analyses. No statistically significant correlation was found between TID and TTD discrimination thresholds (*r* = 0.31, *p* = 0.067). Some evidence of a correlation was found between tactile detection thresholds (measured during screening) for TTDs (*r* = 0.36, *p* = 0.032) but not for TIDs (*r* = 0.17, *p* = 0.309). Two-sampled *t*-tests revealed evidence that TID discrimination thresholds were lower for male (average threshold 0.97 dB) compared to female (average threshold 1.18 dB) participants (*t*(34) = 2.22, *p* = 0.033). Males were around 10 ms more sensitive to TTDs on average (29.80 ms compared to 39.74 ms), but strong evidence of a sex difference was not found (*t*(34) = 0.79, *p* = 0.083).

### Discussion

In experiment 1, the tactile system was shown to be highly sensitive to across-wrist intensity differences. This sensitivity was similar to auditory sensitivity to IIDs. Nearly half of the participants achieved TID discrimination thresholds of less than 1 dB, which is the average IID detection threshold for a 1-kHz tone in young adults^25^. In addition to the finding of high sensitivity to TIDs, no evidence of a decline in sensitivity with age was found for participants up to 60 years old. This is highly encouraging as the vast majority of CI users are below 60 years of age^42,43^. These findings suggest that haptic sound-localisation to within a few degrees^44^ may be obtainable for people across a wide age range.

In contrast to findings for TID discrimination, across-wrist TTD sensitivity was found to be much poorer than auditory sensitivity to ITDs. The best performing participant was able to discriminate TTDs of 10 ms. This is several orders of magnitude worse than sensitivity to ITDs, which is around 10 μs on average for normal-hearing listeners^34^. ITD cues therefore cannot be directly transferred via haptic stimulation. This strongly suggests that in previous haptic sound-localisation studies^1,6^, sounds were localised by exploiting across-wrist TIDs. In addition to sensitivity to TTDs being low relative to ITD sensitivity, it was also found to decline with age. This reduces the potential utility of exploiting TTDs across the wrists in applications involving older individuals.

The observed robustness to aging of TID but not TTD sensitivity is consistent with findings for the auditory system, where IID sensitivity is robust to aging, but ITD sensitivity is not^45^. Our findings are also in broad agreement with previous work on simultaneity detection across the hands^30^, although we found a reduced effect of aging and greater sensitivity to time differences. This may be due to the higher sensitivity of the method used in the current study^31–33^, the different age groups used, and the different stimulation site. The reduced TTD sensitivity with age is also in line with a broader literature suggesting reduced tactile and auditory temporal acuity for older adults^46–48^*.* The reduction in TTD sensitivity is likely to be due to physiological changes in the tactile system. For example, with increasing age, reductions in the density of mechanoreceptors and neural conduction speed have been found, as well as changes in the morphology (twisting) of mechanoreceptors^49–52^*.*

In secondary analysis, some evidence was found that TTD and TID discrimination thresholds differ depending on the sex of the participant. One previous study found that tactile sensation magnitude grows slightly more quickly in women than in men^52^. Another study found that men were more sensitive to TTDs across the fingers than women^29^. In the latter study, it was argued that this difference may be related to the greater volume of white matter in male compared to female brains^53^, indicating more myelinated axons and therefore more rapid signal transfer between brain regions. From this work, it may be expected that TID and TTD discrimination thresholds would be worse in women than in men. In line with this expectation, we found some evidence of lower TID and TTD sensitivity in females, but further work is required to establish the robustness of these effects. Note that there were fewer males in the young age group than females, but an equal number of males and females in the older group. This may have led to an underestimate of male performance, particularly for TTD discrimination thresholds, which were found to worsen with age.

## Experiment 2: level, stimulation type, and dynamic range

### Introduction

Experiment 1 showed that the tactile system is highly sensitive to across-wrist TIDs but not TTDs, and that this TID sensitivity is robust to aging. The first aim of experiment 2 was to establish whether the high sensitivity to across-wrist TIDs is also observed for amplitude-modulated stimuli. Studies of auditory sensitivity to IIDs suggest that sensitivity is higher for amplitude modulated tones than for unmodulated tones^54,55^. Similar, or perhaps higher, TID sensitivity might therefore be expected for amplitude modulated stimuli. The second aim of experiment 2 was to establish how across-wrist TID sensitivity changes with the overall intensity of the stimulus. The auditory system becomes more sensitive to IIDs as sound intensity increases^56,57^. It might therefore be expected that greater across-wrist TID discrimination will be observed for more intense stimuli. Finally, the third aim was to establish the usable dynamic range available at the wrist. For haptic stimulation based on audio extracted from behind-the-ear devices, a dynamic range greater than approximately 15 dB would be required to accommodate direct transfer of IID cues for sounds from all possible angles^6^. The dynamic range available through electrical CI stimulation is around 15 dB^10,11^ and so, if a dynamic-range greater than 15 dB is available through haptic stimulation, additional stimulus intensity information might also be provided.

In experiment 2, tactile detection thresholds and TID discrimination thresholds were measured on the palmer surface of the wrists. TID discrimination was measured for a 250-Hz sinusoid that was either unmodulated or modulated by the amplitude envelope of a speech signal. Measurements were made at 15, 20, 25, and 30 dB above the participant’s detection threshold (dB sensation level (SL)).

### Results

**Figure 2:**
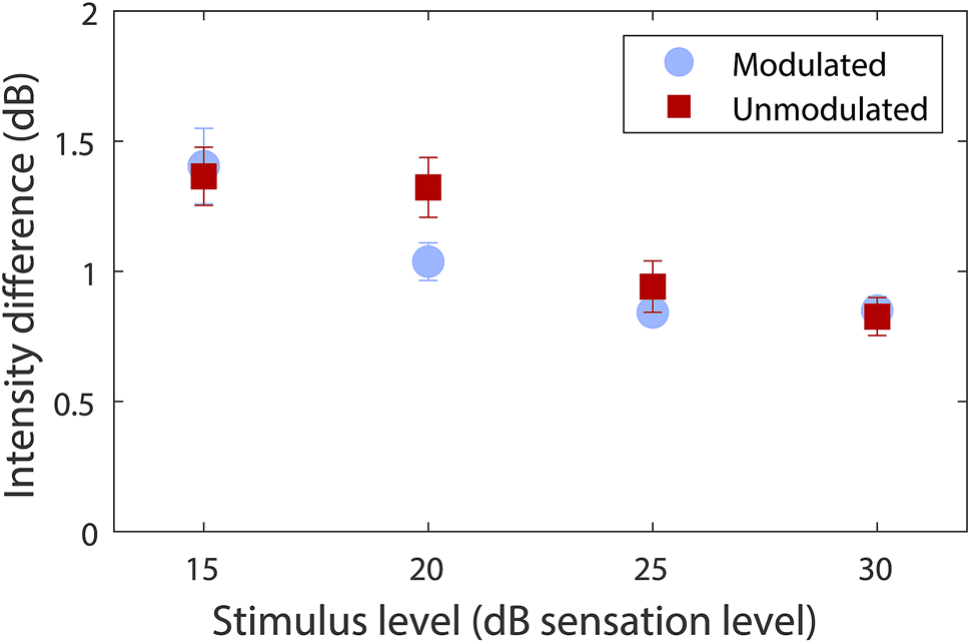
Tactile intensity difference discrimination thresholds across the wrists for speech amplitude envelope modulated (light blue circles) and unmodulated (dark red squares) sinusoids, in young adults. Thresholds are shown as a function of stimulus level above the participant’s detection threshold (sensation level). Error bars show the standard error of the mean. This figure was generated using MATLAB R2019a (http://www.mathworks.com/products/matlab/).

Figure 2 shows TID discrimination thresholds for modulated and unmodulated sinusoids at different stimulus intensities. No significant effect of stimulus type (modulated or unmodulated) was found (*F*(1,15) = 2.39, *p* = 0.143). TID discrimination thresholds averaged across all stimulus intensities were 1.04 dB for the modulated stimulus (ranging from 0.69 to 1.58 dB; SD = 0.23 dB) and 1.12 dB for the unmodulated stimulus (ranging from 0.68 to 1.78 dB; SD = 0.28 dB). A highly significant effect of stimulus intensity was found (*F*(3,45) = 26.54, *p* =  < 0.000). For the modulated stimulus, TID discrimination thresholds reduced from 1.41 dB at 15 dB SL (ranging from 0.47 to 3.12 dB; SD = 0.58 dB) to 0.85 dB at 30 dB SL (ranging from 0.53 to 1.28 dB; SD = 0.22 dB). For the unmodulated stimulus, TID discrimination thresholds reduced from 1.37 dB at 15 dB SL (ranging from 0.62 to 2.44 dB; SD = 0.45 dB) to 0.83 dB at 30 dB SL (ranging from 0.37 to 1.40 dB; SD = 0.29 dB). No interaction between stimulus type and stimulus intensity was found (*F*(3,45) = 1.39, *p* = 0.257).

Post-hoc planned paired-sample *t*-tests, with Bonferroni-Holm correction for multiple comparisons applied^58^, showed no significant difference in TID discrimination thresholds between stimulus types at any stimulus intensity (15 dB SL: *t*(15) = 0.23, *p* =  > 0.999; 20 dB SL: *t*(15) = -2.16, *p* = 0.188; 25 dB SL: *t*(15) = -1.09, *p* = 0.873; 30 dB SL: *t*(15) = 0.31, *p* =  > 0.999). There was also no significant difference in detection thresholds between stimulus types (*t*(15) = 1.583, *p* = 0.670). For the modulated stimulus, average detection thresholds for the left and right wrists were 0.0070 ms^-2^ (with frequency-weighting applied following BS-6842:1987^59^; ranging from 0.0008 to 0.0171 ms^-2^; SD = 0.0045 ms^-2^) and 0.0087 ms^-2^ (ranging from 0.0012 to 0.0268 ms^-2^; SD = 0.0071 ms^-2^). For the unmodulated stimulus, average detection thresholds for the left and right wrists were 0.0060 ms^-2^ (ranging from 0.0013 to 0.0157 ms^-2^; SD = 0.0043 ms^-2^) and 0.0059 ms^-2^ (ranging from 0.0010 to 0.0146 ms^-2^; SD = 0.0044 ms^-2^).

Further exploratory unplanned post-hoc comparisons were performed (with no multiple comparisons correction applied) to assess effects of sex. No difference in TID discrimination threshold was found between males and females for either the modulated (*t*(7) = -0.902, *p* = 0.394) or unmodulated (*t*(7) = 0.203, *p* = 0.845) stimulus. There was also no sex difference in detection thresholds for either stimulus type (modulated: *t*(7) = 1.653, *p* = 0.142; unmodulated: *t*(7) = -0.288, p = 0.782). Finally, a Pearson’s correlation revealed no clear relationship between detection threshold and TID discrimination threshold (averaged across all stimulus intensities) for either the unmodulated (*r* = 0.32, *p* = 0.224) or modulated (*r* = 0.12, *p* = 0.664) stimulus.

### Discussion

In experiment 2, the high sensitivity to across-wrist TIDs found in experiment 1 was shown to apply across a range of stimulus intensities, with sensitivity highest for more intense stimuli. This is consistent with auditory sensitivity to IIDs, which also increases at higher intensities^56,57^. At the highest stimulus level (30 dB SL), an average TID discrimination threshold of 0.83 dB was found for the unmodulated stimulus. Thirteen of the sixteen participants (80%) had thresholds lower than 1 dB (the average sensitivity to IIDs in young adults^25^). This suggests that across-wrist TID discrimination is similar, or perhaps even better, than IID discrimination.

No difference between TID discrimination thresholds for the modulated and unmodulated stimuli was found. This finding contrasts with evidence that IID discrimination is better for amplitude modulated tones than for unmodulated tones^54,55^. It is not clear why there would be differences in amplitude modulated and unmodulated tones for auditory but not tactile stimulation. However, it should be noted that, unlike in the current experiment, comparisons for auditory stimuli were made across different groups of participants. Future work is required to confirm that, for auditory stimuli, these differences can be reproduced within a single group of participants.

The detection thresholds measured in experiment 2 allow the usable dynamic range available for a wrist-worn haptic device to be estimated. To do this, we calculated the difference between the average detection threshold and a safe peak exposure^60^ (assuming that participants wouldn’t be exposed at the peak intensity for more than 2 h per day). This produced an estimated average usable dynamic range of 60 dB, which is around four times larger than the dynamic range available through electrical CI stimulation^10,11^.

## General discussion

In both experiments, remarkably high sensitivity to TIDs was found. For a stimulus at 30 dB SL, participants were able to discriminate TIDs of 0.83 dB on average. The conversion of this to a change in sound location is complicated because the correspondence between sound location and IID varies markedly with both lateralisation and frequency^44^, as well as with the specific haptic signal-processing strategy used. One haptic signal-processing strategy proposed by Fletcher et al.^6^, which is similar to that used in other haptic sound-localisation studies^1,2^, extracts the speech amplitude envelope from signals received by behind-the-ear devices and delivers them to each wrist. Using this approach, a TID of 0.83 dB for a speech signal would correspond to an azimuth change of ~ 3° for sounds located between ± 15° (with 0° corresponding to directly in front of the listener), of ~ 4.5° for sounds between 15° and 30° to the side, and of ~ 7° for sounds between 30° and 45° to the side.

Unlike in some studies of IID discrimination, in which participants were trained for several hours^25^, no training was provided in the current study. Previous studies of IID discrimination have found large improvements in threshold with training over several days^54^. This raises the possibility that the tactile system is capable of even higher sensitivity to across-wrist TIDs than was shown. Extensive training may also be required to fully exploit TID sensitivity for haptic sound-localisation. This suggestion is supported by previous work showing improvements in haptic sound-localisation with training^1,6^ and similar findings of substantial training effects for enhancement of speech-in-noise performance with electro-haptic stimulation^4,7^.

The current study explored the usable dynamic range for haptic stimulation at the wrists. The estimated 60-dB dynamic range was based on a stimulation frequency of 250 Hz and the use of a circular contactor with a 10-mm diameter. These were selected to match the shape and size of the contactor and characteristic frequency of many widely available actuators (such as those used in the Tasbi wrist-worn device^12^). Tactile sensitivity is known to decrease as frequency decreases below 250 Hz. For a stimulation frequency of 100 Hz (the lowest operating frequency typically found in micromotors), tactile sensitivity is reduced by around 15 dB^61^. Detection thresholds are also known to increase with decreasing contactor size, with an approximately 3-dB worsening in threshold for each halving in contactor area^41,62^. For smaller contactor sizes and different characteristic frequencies, the usable dynamic range would therefore be expected to be smaller than estimated in the current study. However, even for small motors with low characteristic frequencies, the dynamic range available is still likely to be substantially larger than the around 15 dB available through electrical CI stimulation. The authors are not aware of studies examining the effect of contactor size on TID discrimination, but it has been observed that subjective magnitude growth is similar for different contactor sizes on the hand^63^. This could suggest that TID discrimination thresholds will not be heavily influenced by contactor size. However, further work is required to establish this.

Another factor that is known to influence tactile detection thresholds is the pressing force of the contactor upon the skin. Detection thresholds are known to improve with increased pressing force^64^, but it is not known whether pressing force also affects TID discrimination. However, modern haptic motor drivers can adjust the driver output based on the back-EMF from the motor in order to correct for differences in pressure applied^65^. Further work is required to understand the impact of the pressing force on TID discrimination across the wrists and how well these effects can be controlled for using modern techniques deployed in haptic drivers.

In the current study, we focused on haptic stimulation applied to the wrists. The wrist was considered a suitable site as wrist-worn devices typically do not impede everyday tasks, do not have impractically restrictive size and weight requirements, are easy to self-fit, and are socially acceptable. One potential drawback of the wrists is that, in real-world settings, their position relative to the body frequently changes. In haptic sound-localisation studies, the haptic signal was extracted from audio received by behind-the-ear hearing-assistive devices^1, 6^; the haptic stimulation being applied to each wrist would therefore not be influenced by changes in arm position. Nonetheless, it is possible that movement, and particularly crossing of the wrists, will lead to confusions that reduce haptic sound-localisation accuracy. An alternative approach is to extract the haptic signal from microphones mounted on each wrist-worn device. An advantage of this approach is that it would allow the user to scan the auditory scene by moving their arms and to direct the microphone towards a talker or other sound source of interest. However, unwanted distortion of haptic sound-localisation cues may be caused by everyday arm movements, such as when walking, cooking, or gesticulating. Effects of arm movement would not have been observed in the current study or previous haptic sound-localisation studies as free movement of the wrists was not permitted. Previous studies have explored the effects of relative hand position on haptic intensity perception. Haptic stimulation on one hand has been shown to modulate haptic intensity perception on the other, but no dependence of this modulation on relative hand position has been found^66^*.* In contrast, it has been shown that temporal-order judgement thresholds worsen when the hands are crossed^67,68^, suggesting that perception of TTDs may be influenced by hand position. It will be important for future work to assess whether changes in wrist position reduce haptic sound-localisation accuracy and whether any negative effects can be reduced with training. It should be noted, however, that even quite crude haptic sound-localisation may be beneficial to many CI users^1,3^ and that motion of the arms may be substantially reduced in other suggested applications, such as robotic and surgical guidance.

In addition to aiding sound localisation, a recent study with CI users has shown that providing haptic stimulation derived from audio received by behind-the-ear devices can improve segregation of spatially-separated sounds^2^. The haptic signal was delivered to each wrist, with frequency information provided by modulating carrier tones with speech amplitude envelopes extracted from four frequency bands. Using this approach, substantial improvements in speech-in-noise performance for spatially-separated speech and noise were found. Other studies have provided frequency information through differences in location of stimulation on the skin^8^. In the current study, frequency information was not provided, though high sensitivity to TIDs was shown for both low and high frequency tactile stimulation. Further work is required to optimise this approach to maximise the benefit of haptic stimulation for separating sound sources.

As well as enhancing listening, high sensitivity to across-wrist TIDs might be exploited for other clinical applications. One example is providing body orientation information to aid those with balance disorders. Another is providing information about the orientation of medical tools to aid those performing high-precision tasks, such as needle steering in brachytherapy^17^. High TID sensitivity could also be exploited in various human–machine interfaces where spatial awareness is critical. For example, human–robot teams commonly deploy robots with numerous sensors to access areas that are too small, deep, or dangerous to be accessed by humans^69^. Haptic stimulation could provide important robot orientation information when visibility is poor or could be used to deliver spatial information about temperature or roughness of terrain. Other potential applications might be found in consumer technology, such as virtual or augmented reality headsets where spatial awareness and immersion are often key. This might include future iterations of a new wrist-worn haptic device developed by Facebook Reality Labs, which attempts to increase immersion in virtual and augmented reality^12^ but currently does not exploit across-wrist cues to deliver spatial or other information.

In this study, remarkably high across-wrist sensitivity to TIDs was found across a range of stimulus intensities and for both amplitude modulated and unmodulated signals. This sensitivity was found to be highly robust to aging. It was also shown that the usable dynamic range for haptic stimulation at the wrist is much larger than that available through electrical CI stimulation. Collectively, these findings suggest that the tactile system has the properties required to provide high-resolution sound localisation and stimulus intensity information to hearing-impaired listeners across a large age range. Given the substantial recent developments in high-fidelity, compact haptic driver and motor technology, as well as advances in microprocessor and battery technology, the time seems right to exploit wearable haptic technology to aid the hearing impaired.

## Methods

### Participants

In experiment 1, two groups of participants were recruited, one young (aged between 20 and 30 years, mean age 26) and one older (aged between 50 and 60 years, mean age 54). The young group contained twenty-two participants (8 male, 14 female) and the older group contained fourteen participants (7 male, 7 female). In experiment 2, sixteen participants (8 males and 8 females, aged between 21 and 33 years, with a mean age of 25) took part. For both experiments, participants were recruited from the staff and students of the University of Southampton, and from acquaintances of the researchers. Informed consent was obtained from all participants, who were paid £10 per hour for taking part. All participants reported no touch perception issues and had vibrotactile detection thresholds within the normal range (see Procedure), indicating no touch perception issues.

### Stimuli

In experiment 1, a sinusoidal vibrotactile stimulus was presented with a frequency of either 100 or 250 Hz. The stimulus had a duration of 200 ms and was ramped on and off with 20-ms raised-sine and -cosine ramps. For the TID measurements, observation intervals were separated by a gap of 200 ms and, for TTD measurements, intervals were separated by 400 ms. The stimulus was set to a nominal level of 1.3 ms^-2^ (frequency-weighted according to the weighting defined in BS-6842:1987^59^), which was determined in piloting to be a comfortable level. For the TID discrimination threshold measurements, the level of each stimulus generated was roved randomly around the nominal level by ± 2 dB (with a uniform distribution) to preclude participants from using single-wrist intensity cues.

**Figure 3:**
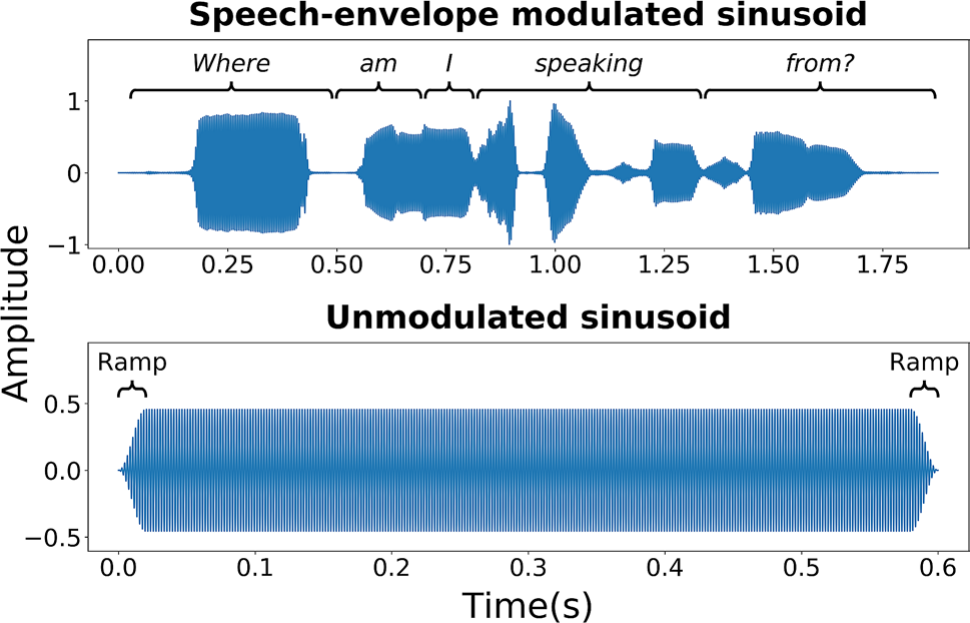
The amplitude envelope (plotted in arbitrary linear units) of the stimuli used in experiment 2. The top panel shows the speech amplitude envelope modulated stimulus (with the sentence text marked above) and the bottom panel shows the unmodulated stimulus (with onset and offset ramps marked above). Both stimuli were sinusoids with a frequency of 250 Hz. This figure was generated using Matplotlib 3.1.3 (https://matplotlib.org/).

In experiment 2, a 250-Hz sinusoidal stimulus was presented, either with or without amplitude modulation applied. The modulated sinusoid had its amplitude modulated by the amplitude envelope of the female speech sample used by Fletcher, et al.^1^ (see Fig. 3). The amplitude envelope was extracted using a 500^th^-order zero-phase FIR filter with a cut-off frequency of 50 Hz. The speech sample is available through the University of Southampton’s Research Data Management Repository at: https://doi.org/10.5258/SOTON/D1206. The unmodulated stimuli had a duration of 600 ms and the modulated stimuli had a duration of 1877 ms (matching the duration of the speech sample). Both stimuli were ramped on and off with 20-ms raised-sine and -cosine ramps. Observation intervals were separated by a gap of 200 ms, as in experiment 1. The intensity of the modulated and unmodulated stimulus was matched for RMS. For the TID discrimination threshold measurements, the stimulus intensity was nominally set to either 15, 20, 25, or 30 dB above the detection threshold for the least sensitive wrist. As in experiment 1, the level of each stimulus was randomly roved around the nominal level by ± 2 dB. For both experiments, all stimuli had total harmonic distortion of less than 0.1% and white noise at a level of 65 dB SPL (A-weighted) was delivered to both ears to mask any audio cues from the shakers.

### Apparatus

In the screening phase, vibrotactile thresholds were measured using a HVLab Vibrotactile Perception Meter^70^ with a 6-mm circular contactor, a rigid surround, and a constant upward force of 2 N (following International Organization for Standardization specifications^71^). This system was calibrated using a B&K calibration exciter (Type 4294).

**Figure 4:**
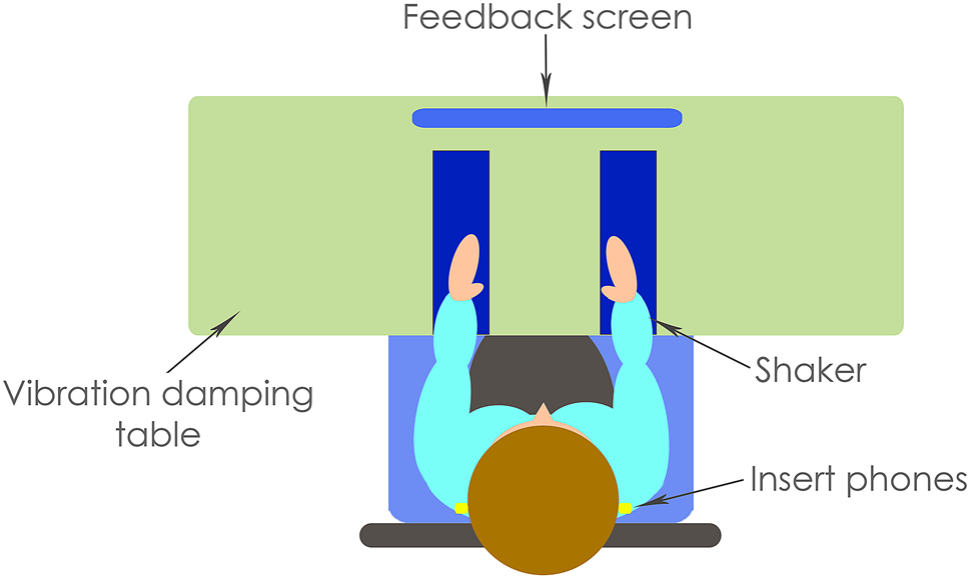
Schematic representation of the experimental set up (not to scale). This figure was generated using Photoshop CS6 (https://www.adobe.com/uk/products/photoshop.html).

For detection threshold measurements in the testing phase, stimuli were generated and controlled using a custom MATLAB script (version R2019a, The MathWorks Inc., Natick, MA, USA). For all other measurements, stimuli were generated and controlled using custom Max 8 (version 8.0.8, Cycling '74, Walnut, CA, USA) patches. Both audio and haptic signals were played out via an RME Babyface Pro soundcard (Haimhousen, Germany; sample rate of 48 kHz and bit depth of 24 bits). Audio was presented using ER-2 insert earphones (Etymotic, IL, USA), which were calibrated using a B&K G4 sound level meter with a B&K 4157 occluded ear coupler (Royston, Hertfordshire, UK). Sound level meter calibration checks were carried out using a B&K Type 4231 sound calibrator. Two HVLab tactile vibrometers were placed shoulder-width apart on a foam surface in front of the participant to deliver the vibrotactile signal to the participants’ wrists (see Fig. 4). The vibrometers were adapted by the substitution of the standard 6-mm probe with a 10-mm probe and the removal of the rigid surround. The 10-mm circular probe matches the contact size used in numerous linear resonant actuators, which are used in haptic devices (e.g. the Tasbi^12^), and the removal of the rigid surround increased the area of skin excitation to more closely match stimulation through a haptic device. A B&K type 4294 calibration exciter was used to calibrate the accelerometers built into the HVLab vibrometers, and vibrometers were calibrated to provide equal amplitude across frequency. Intervals were visually cued and feedback was given using a iiyama ProLite T2454MSC-B1AG 24-inch monitor, placed in front of the participant.

### Procedure

For both experiments, sessions contained a screening and testing phase. In the screening phase, participants first completed a screening questionnaire to ensure that they (1) had no conditions or injuries that may affect their touch perception, (2) had not been exposed to sustained periods of intense hand or arm vibration at any time, and (3) had no recent exposure to intense hand or arm vibration. Following this, participants had their vibrotactile detection thresholds measured at the fingertip to check for normal touch perception. Thresholds were measured following International Organization for Standardization specifications^71^. Participants were required to have touch perception thresholds in the normal range (< 0.4 ms^-2^ RMS at 31.5 Hz, and < 0.7 ms^-2^ RMS at 125 Hz^71^). Finally, otoscopy was performed to ensure insert earphones could be used safely. If the participant passed all screening stages, they moved to the testing phase.

For the TTD trials in the testing phase of experiment 1, the stimuli were presented in a two-reference, two-alternative forced-choice task. On each trial, the stimuli were presented in four observation intervals. The first and fourth intervals contained reference stimuli, with no TTD. The TTD for each trial was applied to the stimulus in either the second or third interval (with equal a priori probability). The participant’s task was to tell the experimenter which of these two intervals had the TTD applied.

For the TID trials in experiment 1, the stimuli were presented in a two-alternative forced-choice task. On each trial, the stimuli were presented in two observation intervals. A TID with higher intensity on the left or right wrist (with equal a priori probability) was applied to the stimulus in the second observation interval. The participant’s task was to tell the experimenter whether the stimulus in the second interval was to the left or the right of the stimulus in the first interval. For both TTD and TID discrimination threshold measurements, intervals were visually cued and feedback on whether the response was correct or incorrect was provided on a visual display.

For TTD and TID discrimination threshold measurements, a three-down, one-up adaptive tracking procedure was used. For TTD discrimination threshold measurements, the starting TTD was 60 ms. TTDs were changed by 10 ms for the first two reversals, by 5 ms for the third reversal, and by 2.5 ms for the remaining six reversals that made up the threshold track. For the TID discrimination threshold measurements, the starting TID was 10 dB. TIDs were changed by 2.5 dB for the first two reversals, by 1 dB for the third reversal, and by 0.25 dB for the remaining six reversals that made up the threshold track. A constant power panning law was used to calculate the gains of each shaker for the TID condition. Panning gains for the shakers were defined as:

$${g}_{1}=\mathrm{cos}\left(\mathrm{arctan}\left({10}^{\left(x/20\right)}\right)\right),$$

$${g}_{2}=\mathrm{sin}\left(\mathrm{arctan}\left({10}^{\left(x/20\right)}\right)\right),$$

where $x$ is the current track value in dB and ${g}_{1}$ and ${g}_{2}$ are the left and right shaker gains in linear units. Positive values of $x$ pan the stimulus to the right and negative values to the left.

For both TTD and TID discrimination threshold measurements, the threshold was defined as the average of the last six reversals. Two threshold tracks were run for each condition, and these were averaged to make the final threshold estimate.

In the experimental phase of experiment 2, detection thresholds were measured at each wrist for the modulated and unmodulated stimuli. A three-alternative forced-choice paradigm was used. Each trial comprised three observation intervals. Only one interval, chosen randomly with equal a priori probability, contained the signal and the participant’s task was to select the interval containing the signal. Intervals were visually cued and visual feedback was given after each trial indicating whether the response was correct or incorrect. The stimulus intensity was varied using a two-down, one-up procedure. The stimulus intensity was initially set at 1.04 ms^-2^ RMS (with frequency-weighting applied following BS-6842:1987^59^; established in piloting to be comfortably above detection threshold) and was changed in 10 dB steps up to the first reversal, in 5 dB steps up to the second reversal, and in 2.5 dB steps for the remaining six reversals. The threshold was defined as the mean of the final six reversals.

Following the detection threshold measurements, participants had a break of at least 15 min before beginning the TID discrimination threshold measurements. TID discrimination thresholds were measured for the modulated and unmodulated sinusoidal stimuli at 15, 20, 25, and 30 dB SL. A two-alternative forced-choice paradigm was used following the method in experiment 1. Two threshold tracks were run for each condition, and these were averaged to make the final threshold estimate.

In experiment 1, the order of conditions (TTD or TID) was fully counterbalanced across participants for each age group, and the order of the frequencies (100 or 250 Hz) was randomised for each repeat. Each experimental session lasted around 1 h. In experiment 2, the order of stimulus types (modulated or unmodulated) was fully counterbalanced across participants, and the order of the levels (15, 20, 25, or 30 dB SL) was randomised for each repeat. Each experimental session lasted around 2 h.

The experimental protocol was approved by the University of Southampton Faculty of Engineering and Physical Sciences Ethics Committee (ERGO ID: 47769). All research was performed in accordance with the relevant guidelines and regulations.

### Statistics

For experiment 1, a mixed analysis-of-variance (ANOVA) with within-subject factor “frequency” (100 or 250 Hz) and between-subject factor “group” (younger or older) was performed on the TID and TTD discrimination threshold data. For experiment 2, data were analysed using a repeated-measures ANOVA, with factors “stimulus type” (modulated or unmodulated) and “stimulus intensity” (15, 20, 25, or 30 dB SL). For both experiments, an alpha level of 0.05 was used.

In experiment 1, unplanned post-hoc two-sample *t*-tests and a Pearson’s correlation were performed for exploratory analysis, with no correction for multiple comparisons applied. For experiment 2, planned post-hoc paired-sample *t*-tests were also conducted to test whether there was an effect of stimulus type on detection thresholds or TID discrimination thresholds at any stimulus intensity. Bonferroni-Holm correction for multiple comparisons was applied. Further unplanned post-hoc comparisons were also performed (with no correction for multiple comparisons applied) to assess effects of sex.

## Data Availability

All data supporting this study are openly available from the University of Southampton repository at 10.5258/SOTON/D1665.
